# MiR-2425-5p targets *RAD9A* and *MYOG* to regulate the proliferation and differentiation of bovine skeletal muscle-derived satellite cells

**DOI:** 10.1038/s41598-017-00470-8

**Published:** 2017-03-24

**Authors:** Hui Li Tong, Run Ying Jiang, Wei Wei Zhang, Yun Qin Yan

**Affiliations:** 1 The Laboratory of Cell and Developmental Biology, Northeast Agricultural University, Harbin, Heilongjiang 150030 China; 2 College of Life Sciences and Agriculture & Forestry, Qiqihar University, Qiqihar, Heilongjiang 161006 China

## Abstract

Our group previously identified miR-2425-5p, a unique bovine miRNA; however, its biological function and regulation in muscle-derived satellite cells (MDSCs) remain unclear. Herein, stem-loop RT-PCR results showed that miR-2425-5p increased during MDSCs proliferation, but decreased during differentiation. Cell proliferation was examined using EdU assays, cyclin B1 (CCNB1) and proliferating cell nuclear antigen (PCNA) western blot (WB) and flow cytometry analysis. These results showed that miR-2425-5p mimics (miR-2425-M) enhanced MDSCs proliferation, whereas, miR-2425-5p inhibitor (miR-2425-I) had opposite effect. Conversely, cell differentiation studies by desmin (DES) immunofluorescence, myotubes formation, and myosin heavy chain 3 (MYH3) WB analyses revealed that miR-2425-M and miR-2425-I blocked and promoted MDSCs differentiation, respectively. Moreover, luciferase reporter, RT-PCR, and WB assays showed that miR-2425-5p directly targeted the 3′-UTR of RAD9 homolog A (*RAD9A*) and myogenin (*MYOG*) to regulate their expression. Rescue experiment showed RAD9A inhibited the proliferation of MDSCs through miR-2425-5p. In addition, we found that miR-2425-5p expression was regulated by its host gene NCK associated protein 5-like (*NCKAP5L*) rather than being transcribed independently as a separate small RNA. Collectively, these data indicate that miR-2425-5p is a novel regulator of bovine MDSCs proliferation and differentiation and provides further insight into the biological functions of miRNA in this species.

## Introduction

MicroRNAs (miRNAs) are a family of endogenous, small-non-coding, functional RNAs. MiRNAs have emerged as key post-transcriptional modulators that bind by complementary base pairing to sequences in the 3′-UTR of target mRNAs^1^, resulting in the repression or degradation of the transcripts. Recently, miRNAs have been implicated in the regulation of myogenic satellite cell proliferation and differentiation. MiRNA-139-5p regulates C2C12 cell myogenesis via Wnt/β-catenin signaling pathway inhibition^2^. MiR-374b directly targets Myf6 and negatively regulates C2C12 myoblast differentiation^3^. MiR-431 promotes the differentiation and regeneration of old skeletal muscle by targeting Smad4 in mice^4^. MiR-675-3p and miR-675-5p can repress Smad1, Smad5, and Cdc6 protein expression to facilitate myogenic differentiation in C2C12 cells^5^. MiRNA-222 regulates Rbm24 alternative splicing during differentiation of skeletal muscle cells in mice^6^. MiR-101a was a positive regulator of goat skeletal muscle satellite cells differentiation^7^. MiR-2400 promotes bovine skeletal muscle satellite cell proliferation by targeting *MYOG* ^8^.

MiR-2425 is a unique bovine *Bos taurus* (cattle) miRNA (NCBI Gene ID: 100313209) expressed in two mature forms: miR-2425-3p and miR-2425-5p. MiR-2425-3p expression was previously reported by Muroya *et al*.^9^, Jevsinek *et al*.^10^, and Romao *et al*.^11^. In the present study, we found that miR-2425-5p is expressed during the proliferation and differentiation of bovine muscle-derived satellite cells (MDSCs) proliferation and differentiation; however, the biological functions of miR-2425-3p and miR-2425-5p remain unknown. Nevertheless, our data suggest that miR-2425-5p may be a novel regulator of MDSCs proliferation and differentiation and warrants further exploration.

Notably, we found that miR-2425-5p binds the 3′-UTR of *RAD9A* and *MYOG* mRNA to downregulate their expression, resulting in enhanced proliferation and attenuated differentiation of bovine MDSCs.

## Results

### miR-2425-5p expression during MDSCs proliferation and differentiation

**Figure 1:**
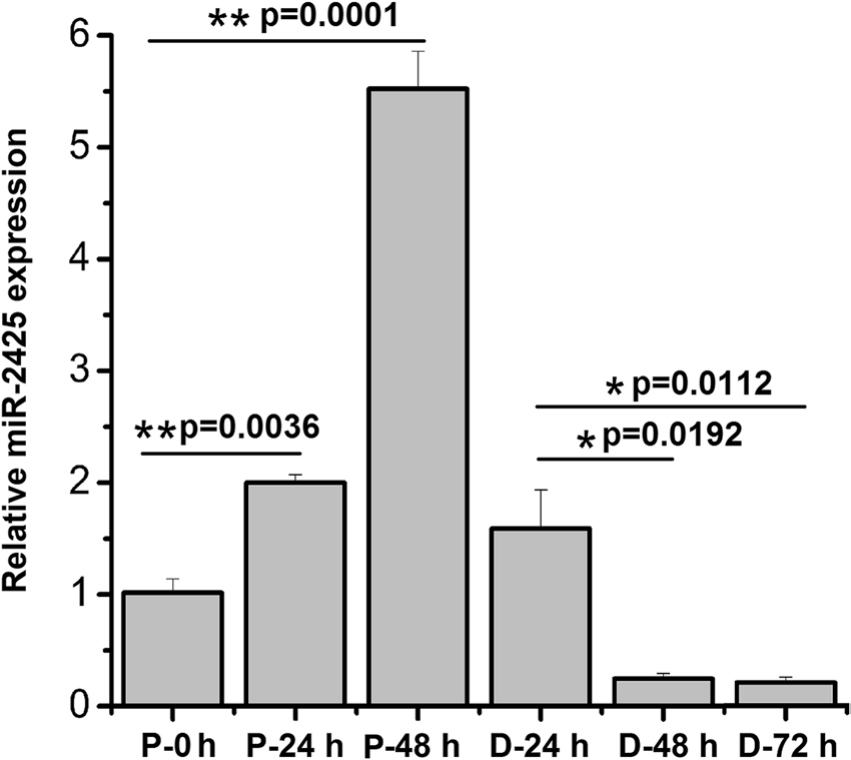
MiR-2425-5p expression during MDSCs proliferation and differentiation. P and D designate proliferating and differentiating cell cultures, respectively. Differentiation was induced with 2% horse serum. Results are shown as the mean ± SEM of three independent experiments. *P < 0.05, **P < 0.01, NS: no significant difference.

The expression levels of miR-2425-5p during the different stages of proliferation and differentiation in MDSCs were detected by stem-loop RT-PCR. The results showed that when compared to non-proliferating cells (P-0 h), miR-2425-5p expression was significantly increased during MDSCs proliferation at 24 h (P-24 h) and 48 h (P-48 h) (P < 0.01), while it decreased during the differentiation. Alternatively, its expression was markedly decreased at 48 h (D-48 h) and 72 h (D-72 h) after the induction of differentiation (P < 0.05) when compared with D-24 h counterparts (Fig. 1). The results of Fig. 1 showed that miR-2425-5p expression reached at its peak at Day 2 (P-48 h) and then reduced towards Day 5 (D-72 h) during the proliferation and differentiation of MDSCs cultured *in vitro*.

### MiR-2425-5p promotes MDSCs proliferation

**Figure 2:**
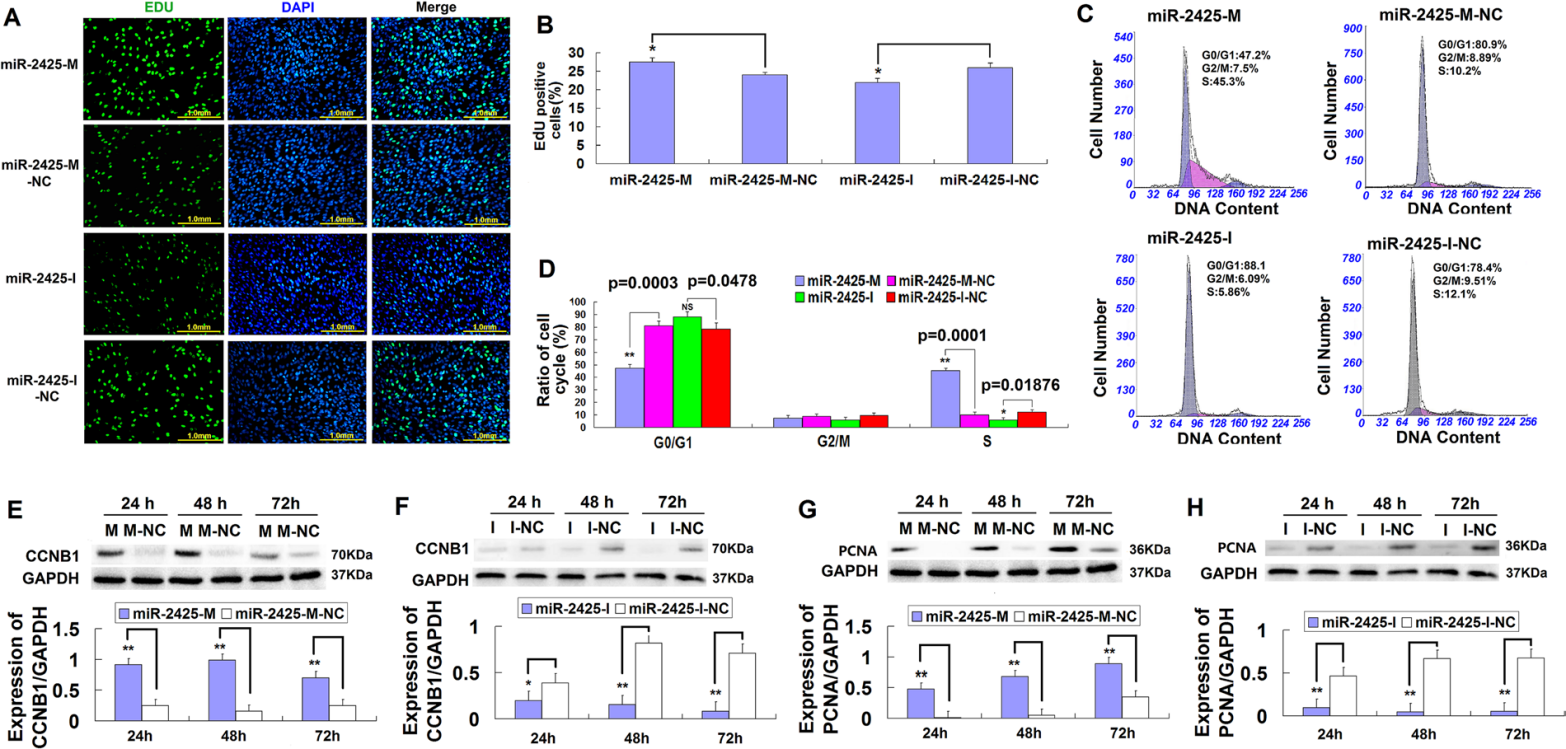
MiR-2425-5p promotes bovine MDSCs proliferation. (**A**) MDSCs were labeled with EdU. EdU-positive cells, green; cell nuclei, blue; magnification, 200×. (**B**) Percentage of EdU-positive cells, n = 6. (**C**) Cycle analysis in cells treated with miR-2425-M, miR-2425-M-NC, miR-2425-I, or miR-2425-I-NC for 48 h. (**D**) Quantification of results shown in (**C**). Data represent the mean ± SEM (n = 3). (E, F) CCNB1 expression in MDSCs treated with (**E**) miR-2425-M or (**F**) miR-2425-I vs. controls at 24 h, 48 h, and 72 h. (**G**,**H**) PCNA expression in MDSCs after treatment vs. controls at 24 h, 48 h, and 72 h. *P < 0.05, **P < 0.01, NS: no significant difference.

Cell proliferation studies showed a significant increase in EdU-positive cells in the miR-2425-M-treated group as compared the miR-2425-M-NC counterparts (p < 0.05), but were substantially decreased in miR-2425-I cells compared to miR-2425-I-NC controls (p < 0.05) (Fig. 2A,B). In addition, differences in cell cycle progression were assessed by flow cytometry after transfection with miR-2425-M, miR-2425-M-NC, miR-2425-I, or miR-2425-I-NC (Fig. 2C). Interestingly, these studies showed that miR-2425-M increased the percentage of cells in S phase and decreased those in G1/G0 phase, whereas miR-2425-I had the opposite effect (Fig. 2D). Moreover, CCNB1 expression was significantly increased in miR-2425-M treated cells at 24 h (p < 0.01), 48 h (p < 0.01) and 72 h (p < 0.01) (Fig. 2E), but significantly decreased with miR-2425-I treatment at 24 h (p < 0.05), 48 h (p < 0.01), and 72 h (p < 0.01) (Fig. 2F). Similar results were observed with PCNA expression, where increased expression was observed at 24 h, 48 h, and 72 h following miR-2425-M treatment as compared to that of miR-2425-M-NC (p < 0.01), while PCNA expression with miR-2425-I treatment decreased at 24 h (p < 0.01), 48 h (p < 0.01), and 72 h (p < 0.01) compared to with miR-2425-I-NC (Fig. 2G,H). Together, these results suggested that miR-2425-5p promotes the bovine MDSCs proliferation.

### MiR-2425-5p inhibits the MDSCs differentiation

**Figure 3:**
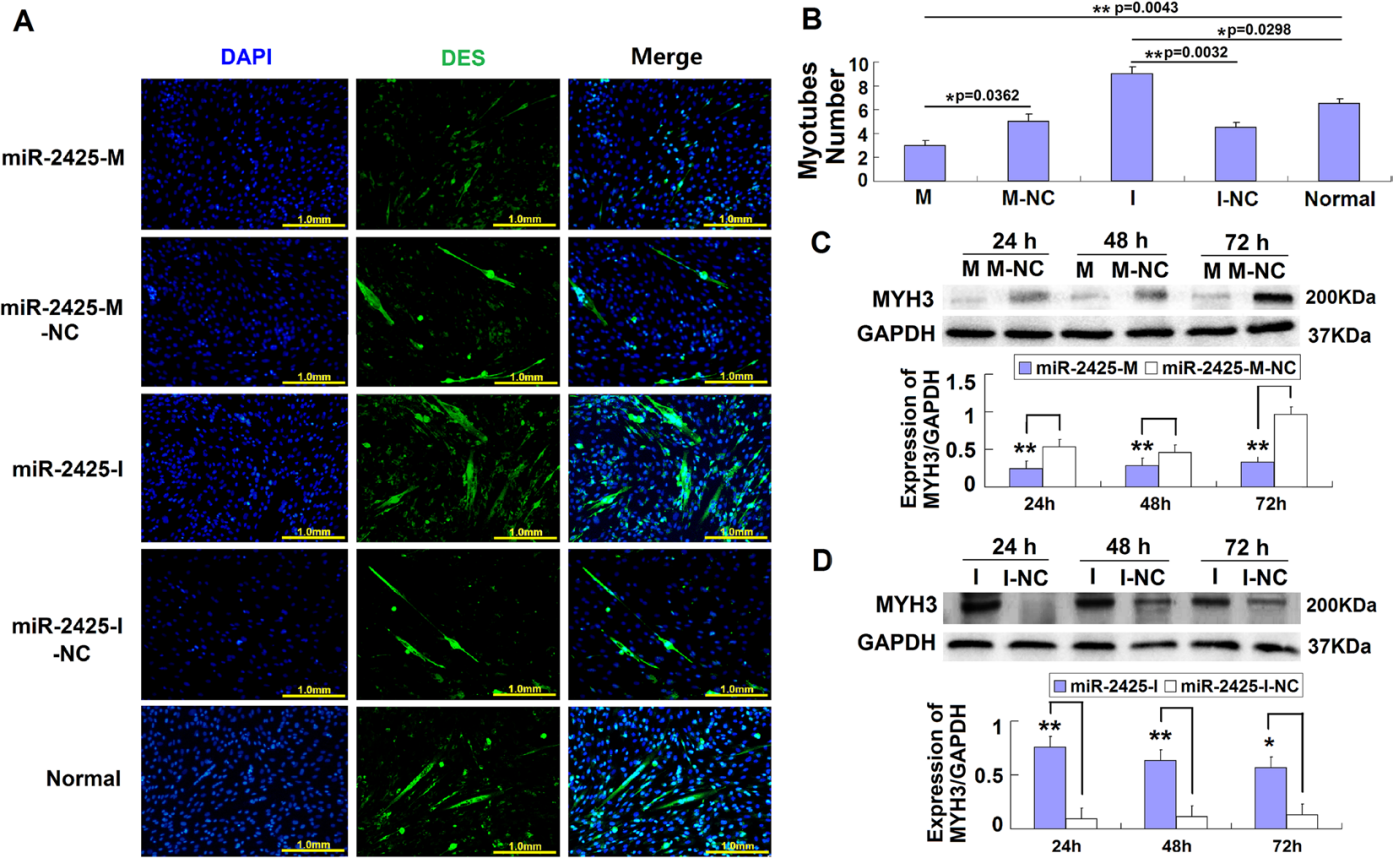
MiR-2425-5p inhibits MDSCs differentiation. (**A**) DES immunofluorescence (green) in MDSCs. The cells were transfected with miR-2425-M, miR-2425-M-NC, miR-2425-I, miR-2425-I-NC, and Normal (MDSCs without any treatment), then differentiated using 2% horse serum for 72 h. Magnification, 200×. (**B**) Quantification of myotubes according to the DES staining presented in A. (**C**) MYH3 expression in MDSCs treated with miR-2425-M vs. controls at 24 h, 48 h, and 72 h. (**D**) MYH3 expression in MDSCs treated with miR-2425-I vs. controls at 24 h, 48 h, and 72 h. *P < 0.05, **P < 0.01, NS: no significant difference.

DES specifically expressed in skeletal muscles. Thus, we used it as a marker of MDSCs differentiation and determined its localization by immunofluorescence. Image analysis showed a significant increase in the number of myotubes at 72 h after treatment with miR-2425-I when compared to that observed with miR-2425-I-NC. Few myotubes were detected after treatment with miR-2425-M compared to miR-2425-M-NC (Fig. 3A). Quantification analysis showed the myotubes number in the miR-2425-M treatment group was significantly reduced when compared to miR-2425-M-NC treated cells (p < 0.05), but were increased in miR-2425-I cultures (p < 0.01), suggesting that miR-2425-5p inhibits the differentiation of MDSCs (Fig. 3B). Moreover, myotubes number in the miR-2425-M group was significantly reduced compared to Normal group cells (without any treatment) (p < 0.01), but was significantly increased in miR-2425-I cultures (p < 0.05).

Furthermore, we also determined that miR-2425-M treatment significantly decreased MYH3 expression during MDSCs differentiation at 24 h (p < 0.01), 48 h (p < 0.01), and 72 h (p < 0.01) (Fig. 3C), while miR-2425-I increased MYH3 expression significantly during MDSCs differentiation at 24 h (p < 0.01), 48 h (p < 0.01), and 72 h (p < 0.05) (Fig. 3D). Together, these results suggested that miR-2425-5p could inhibit the MDSCs differentiation.

### MiR-2425-5p regulates the expression of *RAD9A* and *MYOG*

Target gene prediction for miR-2425-5p revealed 4231 bovine transcripts harboring putative miR-2425-5p binding sites. Among all these transcripts, miR-2425-5p bound the 3′-UTR of *RAD9A* and *MYOG* at 634-640 bp and 437-444 bp, respectively.

**Figure 4:**
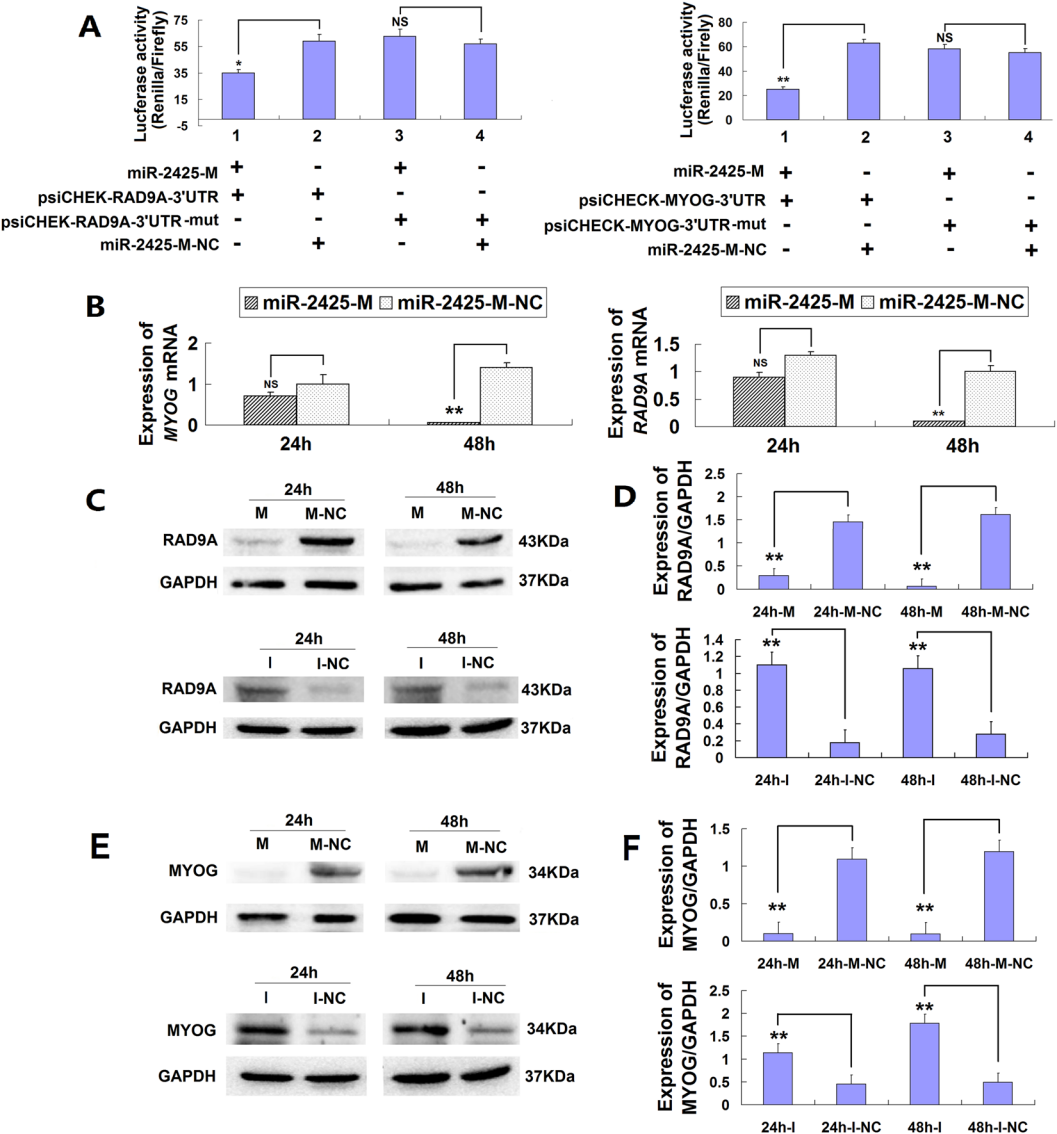
MiR-2425-5p regulates *RAD9A* and *MYOG* expression. (**A**) MiR-2425-5p binding to the 3′-UTR of RAD9A and MYOG was examined with luciferase reporter assays performed by psiCHECK-2 vector. (**B**) *RAD9A* and *MYOG* mRNA expression after miR-2425-M transfection at 24 h, 48 h, and 72 h. (**C**) RAD9A protein expression after transfection of miR-2425-M or miR-2425-I. (**D**) Quantified data from (**C**). (**E**) MYOG protein expression after transfection of miR-2425-M or miR-2425-I. (**F**) Quantified data from (**E**). *P < 0.05, **P < 0.01, NS: no significant difference.

A dual-luciferase reporter system was used to determine the relationship between miR-2425-5p and its target genes, *RAD9A* and *MYOG*. For this, the 3′-UTR sequence and a 3′-UTR mutant sequence within the *RAD9A* and *MYOG* mRNAs were cloned into the psiCHECK expression vector. MiR-2425-M (miR-2425-M-NC) and psiCHECK-RAD9A-3′-UTR (psiCHECK-RAD9A-3′-UTR-mut), miR-2425-M (miR-2425-M-NC) and psiCHECK-MYOG-3′-UTR (psiCHECK-MYOG-3′-UTR-mut) were co-transfected into MDSCs respectively. Luciferase analysis showed that the activities of psiCHECK-RAD9A-3′-UTR (p < 0.05) and psiCHECK-MYOG-3′-UTR were significantly decreased when compared with that of control (p < 0.01) (Fig. 4A), whereas that of psiCHECK-RAD9A-3′-UTR-mut and psiCHECK-MYOG-3′-UTR-mut were not markedly different from that of the control group (Fig. 4A). SYBR Green Quantitative RT-PCR studies showed miR-2425-M could significantly suppress *RAD9A* and *MYOG* endogenous mRNA expression at 48 h (Fig. 4B). WB was also performed to confirm these effects on RAD9A and MYOG at the protein level. As expected, miR-2425-M significantly decreased the RAD9A and MYOG protein expression at 24 h and 48 h (p < 0.01), whereas miR-2425-I significantly increased RAD9A expression at 24 h (p < 0.01) and 48 h (p < 0.01) (Fig. 4C,D). Moreover, we also found that the miR-2425-I was also sufficient to significantly increase MYOG expression at 24 h (p < 0.01) and 48 h (p < 0.01) significantly (Fig. 4E,F). Together, these results showed that miR-2425-5p regulates the RAD9A and MYOG expression by directly targeting their 3′-UTR.

### RAD9A inhibits the MDSCs proliferation through miR-2425-5p

**Figure 5:**
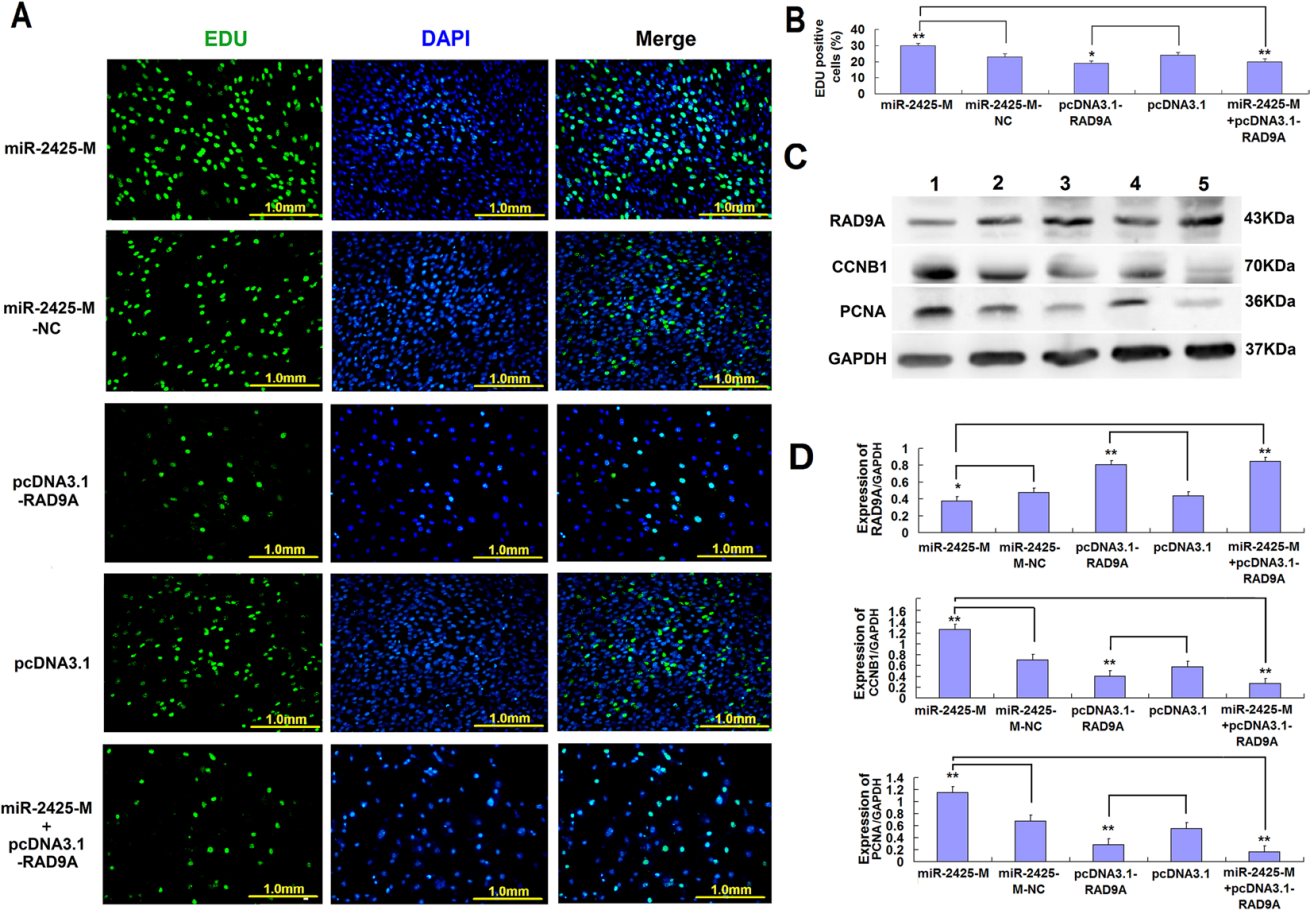
Results of RAD9A rescue experiment. (**A**) MDSCs were labeled with EdU. EdU-positive cells, green; cell nuclei, blue; magnification, 200×. (**B**) Percentage of EdU-positive cells, n = 6. (**C**) RAD9A, CCNB1, and PCNA protein expression was examined 48 h after transfection with the following constructs: (1) miR-2425-M, (2) miR-2425-M-NC, (3) pcDNA3.1-RAD9A, (4) pcDNA3.1, (5) miR-2425-M + pcDNA3.1-RAD9A. (**D**) Quantified data shown in (**C**). *P < 0.05, **P < 0.01, NS: no significant difference.

In rescue experiments, exogenous RAD9A and miR-2425-M were co-transfected into MDSCs. Interestingly, these results showed that miR-2425-M significantly increased the number of EdU-positive cells when compared with miR-2425-M only controls (p < 0.01), while RAD9A overexpression alone decreased the number EdU-positive cells compared with pcDNA3.1 empty vector controls (p < 0.05). Moreover, when miR-2425-M was combined with pcDNA3.1-RAD9A, the number of EdU-positive cells decreased significantly compared with miR-2425-M only controls (p < 0.01) (Fig. 5A,B). As expected, WB results showed that pcDNA3.1-RAD9A transfection increased RAD9A expression in MDSCs, but resulted in a downregulation of CCNB1 and PCNA. Similarly, miR-2425-M could decrease RAD9A expression, and subsequently enhanced CCNB1 and PCNA levels when compared with the miR-2425-M-NC group. Further, in rescue experiment group, pcDNA3.1-RAD9A transfection decreased the expression of CCNB1 and PCNA even in the presence of miR-2425-M (Fig. 5C,D), suggesting that RAD9A inhibits MDSCs proliferation via miR-2425-5p.

### MiR-2425-5p co-expression with its host gene, *NCKAP5L*

**Figure 6:**
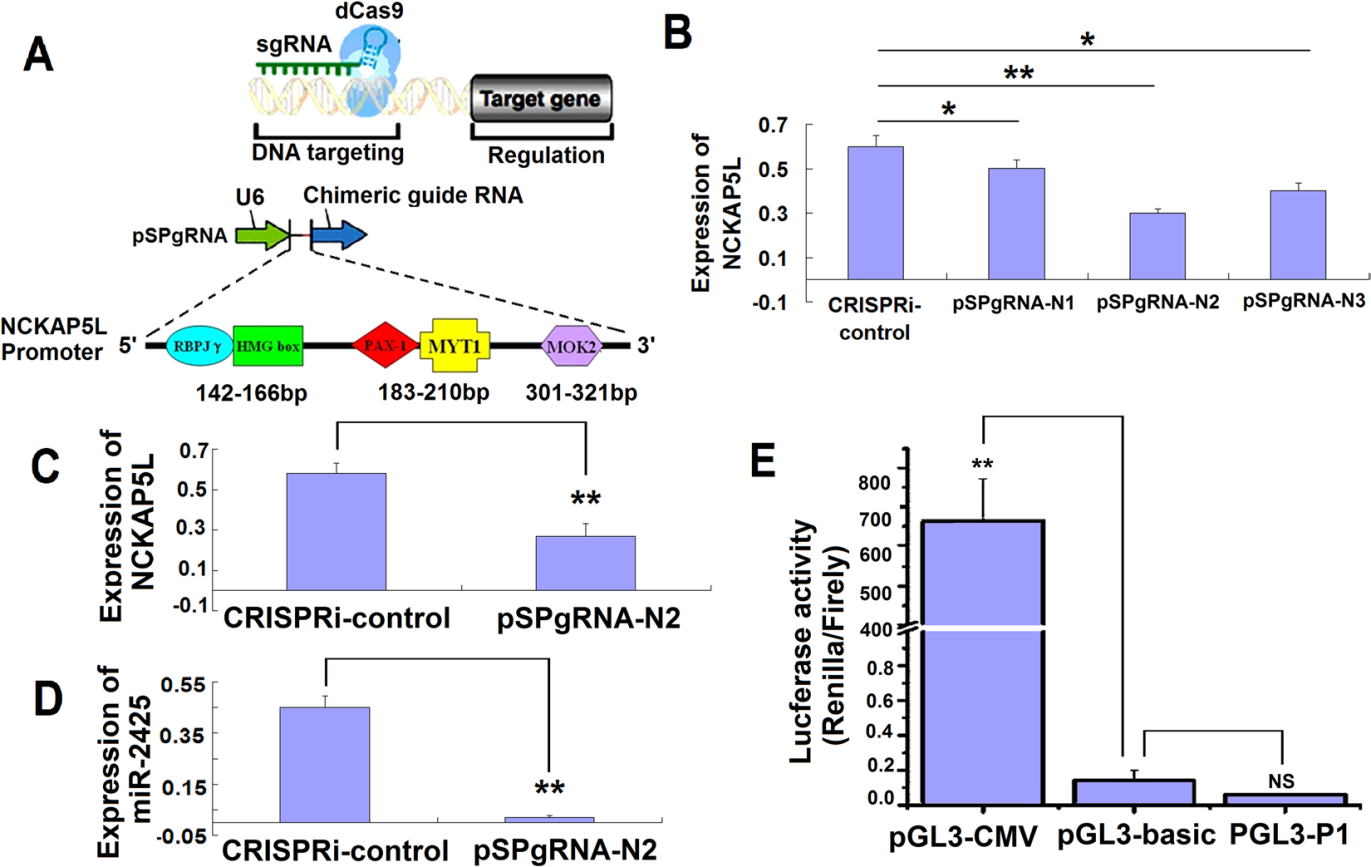
MiR-2425-5p is co-expressed with its host gene, *NCKAP5L*. (**A**) Three sgRNAs targeting sites within the *NCKAP5L* promoter. (**B**) NCKAP5L expressed in MDSCs after co-transfection of the CRISPRi system. pSPgRNA-N1, pSPgRNA-N2 and pSPgRNA-N3 that represented different inhibitory sequences were co-transfected into MDSCs with dCas9. (**C**) NCKAP5L expression after co-transfection with dCas9 and pSPgRNA-N2. (**D**) MiR-2425-5p expression after co-transfection with dCas9 and pSPgRNA-N2. (**E**) Luciferase activity of pGL3-P1 in MDSCs. *P < 0.05, **P < 0.01, NS: no significant difference.

MiR-2425-5p is an intronic miRNA located in intron 1 of *NCKAP5L* (http://www.mirbase.org/). Bioinformatics analyses predicted that some intronic miRNAs are transcriptionally linked to the expression of their host gene^12^, while others exhibit their own transcription regulatory elements, including promoters and terminator signals. The transcription of *NCKAP5L* was repressed by CRISPR interference (CRISPRi) to determine the relationship between miR-2425-5p and *NCKAP5L* expression. For this, three sgRNA targeting sites of the *NCKAP5L* promoter were designed and cloned into a pSPgRNA expression vector (pSPgRNA-N1, pSPgRNA-N2, pSPgRNA-N3) (Fig. 6A). After co-transfection with the dCas9 expression vector into MDSCs, *NCKAP5L* mRNA level was decreased by 81% in the pSPgRNA-N2 group when compared to controls (p < 0.01) (Fig. 6B). SYBR Green Quantitative RT-PCR results showed that miR-2425-5p expression synchronously decreased when *NCKAP5L* expression was decreased by pSPgRNA-N2 (p < 0.01) (Fig. 6C,D). Dual-luciferase reporter assays were used to verify whether miR-2425-5p harbored its own transcription regulatory elements for *NCKAP5L*-independent expression. Co-transfection of pGL3-P1 (P1 was a own promoter sequence within the miR-2425-5p precursor to promote the expression of mature miR-2425, P1 was cloned into pGL3-control vector) with phRL-TK into MDSCs showed that pGL3-P1 luciferase activity was not significantly different with that of the pGL3-control vector, which was significantly lower than that of pGL3-CMV (p < 0.01) (Fig. 6E). Together, these results showed that miR-2425-5p was processed from its host gene *NCKAP5L* rather than being transcribed independently as a separate small RNA.

## Discussion

The purpose of this study was to explore the role of miR-2425-5p in bovine MDSCs proliferation and differentiation. Many studies reported that specific miRNAs play key roles in myogenesis in recent years^13–23^ (Table 1). A deep sequencing on miRNAs expression in bovine MDSCs in our previous report showed that miR-2425-5p expression decreased during MDSCs differentiation at 24 h (−2.82 fold change) and 72 h (−1.82 fold change) compared with MDSCs proliferation at 0 h^24^. Of note, miR-2425-5p appears to be bovine-specific and has not been identified in any other mammalian genomes. Here we show that miR-2425-5p expression was upregulated during MDSCs proliferation at 24 h, 48 h, and downregulated upon differentiation (Fig. 1); thus, we speculated that miR-2425-5p might promote the proliferation and inhibit the differentiation of MDSCs. To assess this hypothesis, we elevated miR-2425-5p levels by exogenous overexpression or synthetic mimics or blocked its activity with a specific inhibitor. Moreover, miR-2425-M-treated cells showed a significant increase in EdU incorporation, as well as a higher percentage of S phase cells. Consistently, these cells also showed a significant upregulation in the proliferation genes *CCNB1* and *PCNA*. In contrast, miR-2425-I had the opposite effect on these characteristics (Fig. 2). These results demonstrated that miR-2425-5p likely facilitates cell proliferation. MDSCs proliferation is an important topic related to muscle regeneration. A previous report found that miR-489 was highly expressed in quiescent satellite cells and quickly downregulated upon cell activation. Subsequent functional studies revealed that miR-489 targeted the oncogene *DEK* to maintain the quiescence in the adult stem-cell population^25^. Similarly, miR-29a targeted *FGF2* to stimulate myoblast proliferation and regeneration of adult skeletal muscle^17^. Our data indicate that miR-2425-5p exhibits a similar expression pattern to that of miR-489 and promotes MDSCs proliferation. While this suggests that miR-2425-5p may play a role in bovine skeletal muscle regeneration, the exact mechanism remains unclear.

**Table 1:** Specific miRNAs with key roles in myogenesis.

miRNAs	Mechanism	Species
miR-29a^13^	Up-regulated MyoD expression and conversely down-regulated Cdk6 expression	Mice
miR-1, miR-206^14^	Positively regulated bovine skeletal muscle satellite cell differentiation via Pax7 and HDAC4	Bovine
miR -128^15^	Regulated the proliferation and differentiation of bovine skeletal muscle satellite cells by repressing Sp1	Bovine
miR-145a-5p^16^	Enhanced or repressed the expression of some special genes involved in the endogenous Wnt signaling pathway	Mice
miR-29a^17^	Controlled skeletal muscle regeneration during injury and exercise downstream of FGF-2	Mice
miR-206^18^	Attenuated denervation-induced skeletal muscle atrophy through regulation of satellite cell differentiation via TGF- b1, Smad3, and HDAC4 signaling	Rats
miR-378^19^	Promoted myogenic differentiation by targeting BMP4	Mice
miR-20a-5p, miR-20b-5p^20^	Involved in myoblast proliferation and differentiation through the auto-regulation with E2F1	Chicken
miR-17-92^21^	Regulated myoblast proliferation and differentiation by targeting the ENH1/Id1 signaling axis	Mice
miR-195, miR-497^22^	Inhibited myoblast proliferation by targeting Igf1r, Insr and cyclin genes	Mice
miR-30-5p^23^	Regulates muscle differentiation and alternative splicing of muscle-related genes by targeting MBNL	Mice

In addition, miR-2425-5p overexpression blocked the MDSCs differentiation as determined by the suppression of muscle-specific differentiation markers *DES* and *MYH3.* DES, the muscle-specific member of the intermediate filament (IF) family, is one of the earliest known myogenic markers in both skeletal muscle and heart^26^. It predominantly expressed in myotubes in bovine skeletal muscle cells^27^. While MYH3 is a major structural protein of the thick filament of the sarcomere, it mainly expressed in the skeletal muscle in different stages, and it plays an important role in the development of skeletal muscle^28^. In this study, miR-2425-5p inhibition resulted in a significant increase in MYH3 protein expression during MDSCs differentiation at 24 h, 48 h, and 72 h (Fig. 3C,D). Moreover, DES staining showed that the number of myotubes—resulting from the fusion of MDSCs—also increased, suggesting that miR-2425-5p expression hinders MDSCs differentiation. Furthermore, myotubes number in the miR-2425-M group was significantly reduced compared to Normal group cells (p < 0.01), but was significantly increased in miR-2425-I cultures (p < 0.05) (Fig. 3A,B). All these results suggested that miR-2425-5p expression hinders MDSCs differentiation.

MiRNAs are known to repress the expression of their target genes by binding to target sequences at specific sites^1^. To investigate the effect of miR-2425-5p on MDSCs proliferation and differentiation, bioinformatics analysis was performed and revealed two target genes, *RAD9A* and *MYOG*. There were two reasons for choosing the two genes. Firstly, according to the prediction results of Target Scan Human 7.0, R*AD9A* and *MYOG* were found in the list of miR-2425-5p target transcripts. Meanwhile, when *RAD9A* and *MYOG* were input into the website of Target Scan Human 7.0, miR-2425-5p binding positions can be found in 3′ UTR of *RAD9A* (position of 634-640 bp) and *MYOG* (position of 437–444 bp) respectively. Another reason was in previous study of our laboratory, *RAD9A* (the fold-changes was −2.40) and *MYOG* (the fold-changes was 3.70) expression changed significantly during MDSCs differentiation at 72 h compared with MDSCs proliferation at 0 h respectively^29^.

*RAD9A* is an evolutionarily conserved gene with multiple functions important for genomic integrity^30^. The roles of mammalian RAD9A in cell cycle checkpoint control, apoptosis, and DNA repair are well established in mitotically dividing cells^31, 32^. As a component of RAD9A-HUS1-RAD1 (9-1-1) complex, RAD9A promotes genomic repair when DNA is damaged^33^. As such, abnormal expression of RAD9A has been linked to tumorigenesis^34^. In comparison, *MYOG* is an important myogenic regulatory factor necessary for myocyte differentiation and the development of functional skeletal muscle^35^.

Notably, our study revealed that RAD9A overexpression decreased the number EdU-positive cells as compared with empty vector controls (p < 0.05), as well as CCNB1 and PCNA expression (Fig. 5), suggesting that RAD9A inhibits MDSCs proliferation. In addition, a rescue experiment was performed to verify that miR-2425-5p/*RAD9A* interaction was necessary to inhibit MDSCs proliferation. For this, the RAD9A CDS (lacking the 3′ UTR sequence) was cloned into the pcDNA3.1 vector (pcDNA3.1-RAD9A). Significantly, miR-2425-M was were unable to block the effects of pcDNA3.1-RAD9A. As expected, miR-2425-M increased the number EdU positive cells but pcDNA3.1-RAD9A decreased the number EdU positive cells compared with their control groups respectively. In addition, miR-2425-M could decrease RAD9A expression, and subsequently enhanced CCNB1 and PCNA levels when compared with the miR-2425-M-NC group. However, pcDNA3.1-RAD9A transfection increased RAD9A protein expression in MDSCs, but resulted in a downregulation of CCNB1 and PCNA. When miR-2425-M and pcDNA3.1-RAD9A were co-transfected into MDSCs, miR-2425-M were unable to block the effects of pcDNA3.1-RAD9A, results in pcDNA3.1-RAD9A still decreased the number EdU positive cells and down-regunalted the expression of CCNB1 and PCNA even in the presence of miR-2425-M (Fig. 5). In another word, RAD9A inhibited the proliferation of MDSCs, miR-2425-M treatment promoted MDSCs proliferation through inhibiting the expression of RAD9A. At the same time, pcDNA3.1-RAD9A was co-transfected with miR-2425-M into MDSCs rescued the RAD9A proliferation inhibitory effect on MDSCs, suggesting that RAD9A inhibits MDSCs proliferation via miR-2425-5p (Fig. 5).

Dual-luciferase reporter assays showed that expression of both the Renilla/Firefly luciferase activities of psiCHECK-RAD9A-3′-UTR and psiCHECK-MYOG-3′-UTR were significantly decreased compared with controls group respectively. It demonstrated miR-2425-5p could directly bind to the 3′-UTR of *RAD9A* and *MYOG* to reduce the expression of psiCHECK-2 dual-luciferase reporter plasmid. To prove this point sufficiently, psiCHECK-RAD9A-3′-UTR-mut and psiCHECK-MYOG-3′-UTR-mut were co-transfected with miR-2425-M into cells respectively. miR-2425-5p can not bind with the mutation sites in 3′-UTR of psiCHECK-RAD9A-3′-UTR-mut and psiCHECK-MYOG-3′-UTR-mut, then it was easy to explain their Renilla/Firefly luciferase activities have no significant difference from that of their control groups (Fig. 4A). In addition, miR-2425-5p significantly suppressed *RAD9A* and *MYOG* endogenous mRNA expression at 48 h (Fig. 4B). WB results showed miR-2425-M significantly decreased the RAD9A and MYOG protein expression, whereas miR-2425-I significantly increased RAD9A and MYOG expression at 24 h and 48 h (Fig. 4C,D,E,F). These results demonstrated that overexpression of miR-2425-5p bind with 3′-UTR of RAD9A and MYOG to down-regulate their mRNA and protein expression respectively. In contrast, miR-2425-I reduced its down-regulate effect to increase the expression of RAD9A and MYOG. As such, these results demonstrated that miR-2425-5p can bind with 3′-UTR of *RAD9A* and *MYOG* and regulate the expression of RAD9A and MYOG.

MiR-2425-5p is located within the first intron of *NCKAP5L*. While the expression and functional aspects of intronic miRNAs are still unknown, it is generally believed that both the host gene and miRNA share a regulatory control system. NCKAP5L is conserved in humans, chimpanzee, Rhesus monkey, dog, mouse, rat, and frog. NCKAP5L was reported in bovine in 2009^36^ and encodes a protein involved in proteolysis and GTPase-mediated signaling^37^. However, very little is known about the biological function of NCKAP5L and it should be investigated in further research.

CRISPRi represents a newly developed tool for targeted gene repression in many organisms. It has great application potential for studying gene function and mapping gene regulatory elements. Meanwhile, dCas9, which lacks endonuclease activity but can still bind to target loci, has been engineered for efficient gene transcription repression^38, 39^. According to this CRISPRi system, we used a designed sgRNA, dCas9-sgRNA complex bound to the specific elements of *NCKAP5L* promoter, complementally induced by sgRNA, to cause a steric block halting the transcript initiation of RNA polymerase, resulting in *NCKAP5L* repression. Notably, pSPgRNA-N2 decreased NCKAP5L expression by 81% compared with that in the control group (Fig. 6B). Interestingly, miR-2425-5p expression was also significantly decreased upon NCKAP5L downregulation (Fig. 6C,D). To confirm this finding, a promoter segment (P1) within the miR-2425-5p precursor was cloned into the pGL3 vector. PGL3-P1 was used in luciferase assays to determine whether miR-2425-5p harbors its own transcription regulatory elements. These results showed that the luciferase activity of this segment was similar with that of the pGL3-control vector (Fig. 6E), indicating that miR-2425-5p lacked its own transcriptional regulatory elements and that its expression was directly linked to its host gene *NCKAP5L*.

In summary, this study suggests that miR-2425-5p is a novel regulator of the bovine MDSCs development. Altogether, these findings highlight the important roles of miR-2425-5p in the maintenance and proliferation of bovine MDSCs. Moreover, while *RAD9A* and *MYOG* were identified as miR-2425-5p target genes, other target genes may also be involved in this process and should be examined in future studies. Nevertheless, miR-2425-5p promoting proliferation and hindering differentiation of bovine MDSCs will be helpful to control the number of skeletal muscle satellite cells and the regulation of miR-2425 will have good prospects in homeostasis of satellite cell population and muscle regeneration.

## Methods

### Ethical statement

The protocol utilized in this study to harvest cells from animal tissues was approved by the Animal Care Commission of the Northeast Agricultural University and Heilongjiang, P.R. China. Skeletal muscle tissues from newborn Chinese Simmental calves were obtained from the Shuangcheng abattoir, a local slaughterhouse in Heilongjiang, P.R. China.

### MDSCs culture and differentiation

MDSCs were isolated from the hind limb muscle tissue of newborn Chinese Simmental calves (n = 3). Skeletal muscle tissues were pooled, finely minced, and digested by treatment with 0.2% collagenase XI (Sigma-Aldrich, St. Louis, MO, USA) combined with 0.25% trypsin (Sigma-Aldrich). The methods used to isolate, purify, and differentiate MDSCs were previously described^25^. MDSCs culture medium was composed of Dulbecco’s modified Eagle’s medium (DMEM, High glucose; Gibco, Grand Island, NY, USA), 20% fetal bovine serum (FBS; Gibco), 10% horse serum (Gibco), 100 IU/mL penicillin, and 100 IU/mL streptomycin (Gibco). Subsequently, the cells were switched to differentiation medium (DM) containing 2% horse serum (Gibco), 100 U/mL penicillin, and 100 μg/mL streptomycin in DMEM.

### Plasmid construction

For RAD9A and MYOG 3′-UTR reporter assay, the entire 3′-UTR of bovine *RAD9A* and *MYOG* were PCR-amplified from bovine genomic DNA and cloned into psiCHECK-2 dual-luciferase reporter plasmid (Promega, Madison, WI, USA) to generate psiCHECK-RAD9A-3′-UTR and psiCHECK-MYOG-3′-UTR. The mutant bovine RAD9A and MYOG 3′-UTR reporters, designated as psiCHECK-RAD9A-3′-UTR-mut and psiCHECK-MYOG-3′-UTR-mut separately, were created by mutating the seed region of the predicted bta-miR-2425 site by nested PCR.

To overexpress RAD9A exogenously, the RAD9A coding sequence (CDS) (NM_001014848.1) was cloned into the pcDNA3.1 vector and designated as pcDNA3.1-RAD9A. 3′-UTR of RAD9A was not cloned in pcDNA3.1-RAD9A. Thus, the overexpression of RAD9A cannot be affected by miR-2425-M.

For miR-2425-5p promoter reporter assay, the intron 1 of *NCKAP5L*, which contains the pre-miR-2425-5p (the sequence named as P1) was PCR-amplified from bovine genomic DNA and cloned into pGL3-control vector (Promega) to generate pGL3-P1. The primers used for plasmid construction were shown in Table 2.

**Table 2:** Primers used for plasmid construction.

gene	Forward primer	Reverse primer
pre-miR-2425	cggggtacctcccttctttacctgactt	ccggaattc cctttgttaccccgactt
psiCHECK-2-RAD9A-3′-UTR	ccctcgagggccctgctttgccctgagctgcag	atgcggccgcagcagcatccagtctccc
psiCHECK-2-RAD9A-3′-UTR -mut	ccctcgagggccctgctttcggctgagctgcagcctta	atgcggccgcagcagcatccagtctccc
psiCHECK-2-MYOG-3′-UTR	ccgctcgagatctgaccaaggtctctgtgctgaagttg	ataagaatgcggccgcctagcacccagtctttattt
psiCHECK-2-MYOG-3′-UTR -mut	ccgctcgagatctgaccaaggtctcacacgtgaagttgc	ataagaatgcggccgcctagcacccagtctttattt
miR-2425-intron of NCKAP5L	caggagaaggtggtgatgg	gataagtgagagaagccccagcagg

### Cell proliferation assay

Cell proliferation was assessed by EdU incorporation and flow cytometry. MDSCs were seeded and transfected with miR-2425-M-NC, miR-2425-M, miR-2425-I-NC, and miR-2425-I. The cells were then maintained in growth medium for 48 h. For EdU incorporation assay, proliferating cells were determined by using the Cell-Light™ EdU Apollo® 488. For quantification analysis, each data point represents the positive fluorescence area calculated from a minimum of five randomly chosen fields from three individual experiments.

Cell cycle flow cytometry was performed 48 h after transfection with miR-2425-M, miR-2425-M-NC, miR-2425-I, or miR-2425-I-NC. Trypsinized cells were fixed in 70% (v/v) ethanol at −20 °C for 12 h. Cells were then incubated in 50 mg/mL propidium iodide solution (100 mg/ml RNase A and 0.2% (v/v) Triton X-100) for 30 min at 4 °C. MDSCs were analyzed on Cytomics^TM^ FC 500 and CXP software (Beckman Coulter, Brea, CA, USA).

### Luciferase reporter assay

Target Scan Human 7.0 (http://www.targetscan.org/vert_71/) was used to predict and analyze miR-2425-5p target genes as previously described^40^.

Luciferase assay was used to determine whether *RAD9A* and *MYOG* were target genes of miR-2425. MDSCs (2.0 × 10^4^ cells per well) were plated in 24-well plates (Corning, Corning, NY, USA) 24 h before transfection for luciferase reporter assay. Both types of cells were transfected with psiCHECK-MYOG-3′-UTR, psiCHECK-MYOG-3′-UTR-mut, psiCHECK-RAD9A-3′-UTR, psiCHECK-RAD9A-3′-UTR-mut, and empty vector psiCHECK-2. The three cell groups were also co-transfected with miR-2425-M. After co-transfection of 48 h, cells were lysed in Passive Lysis Buffer (Promega) and activities of Firefly and Renilla luciferase were measured with a GloMax 20/20 Luminometer (Promega) using the Dual-Luciferase Reporter Assay System according to the manufacturer’s protocol.

### Real-time PCR

Total RNA was extracted from cultured MDSCs using TRIzol (Invitrogen, Carlsbad, CA, USA). Concentrations of total RNA were determined spectrophotometrically using a NanoDrop 2000 C Spectrophotometer (ThermoFisher, Waltham, MA, USA). *RAD9A* and *MYOG* expression was detected by SYBR green quantitative RT-PCR using an ABI7300 Real Time Detection System as previously described^6^. Primer sequences were listed in Table 3. Relative gene expression was determined by the 2^−ΔΔCt^ method. All reactions were performed in triplicate.

**Table 3:** Primers used for SYBR green quantitative RT-PCR.

gene	Forward primer	Reverse primer
miR-2425-5p stem-loop	gcgcgcccagggcacggattccat	gtgcagggtccgaggt
*MYOG*	gactcaagaaggtgaatgaagcc	tattatagtgcgctgccccac
*RAD9A*	cggcttcctcctgttgctg	tcatccaggtctaggtgggg
*GAPDH*	accacagtccatgccatcac	tccaccaccctgttgctgta
*18S*	ggacatctaagggcatcacag	aattccgataacgaacgagact
*ACTB*	gacctctacgccaacacg	gcagctaacagtccgccta

### Western blot analysis

Protein samples were prepared from MDSCs. Briefly, the cells were rinsed twice with ice cold PBS, placed in lysis buffer, and then incubated for 30 min on ice. The cell lysates were centrifuged (12,000 rpm) at 4 °C for 15 min. The resultant samples were resolved by electrophoresis on a 12% SDS-polyacrylamide gel and then transferred to a PVDF membrane (Millipore Corporation, Billerica, MA, USA). The membrane was incubated with a primary antibody MYOG (sc-52903, Santa Cruz Biotechnology, Inc., Dallas, TX, USA), RAD9A (bs-4179, Bioss, Beijing, China), CCNB1 (sc-595, Santa Cruz Biotechnology, Inc.), PCNA (sc-9857, Santa Cruz Biotechnology, Inc.), DES (sc-14026, Santa Cruz Biotechnology, Inc.), and MYH3 (sc-324154, Santa Cruz Biotechnology, Inc.) followed by the addition of a secondary antibody (HRP-labeled goat anti-mouse or rabbit IgG (Santa Cruz Biotechnology, Inc.). The proteins were visualized by Super ECL Plus detection kit (Applygen Technologies Inc., Beijing, China) according to the manufacture's instruction. The membranes were exposed in Mini Chemiluminescent Imaging and Analysis System named MiniChemi™ 500 (Sage Creation Science, Beijing, China) to acquire the image.

### Immunofluorescence

Cells on coverslips were fixed with methanol at −20 °C for 10 min and then washed with Tris buffered saline (TBS). Immunochemical staining was carried out following the manufacturer's instructions. Briefly, cells were incubated for 1 h with TBS containing 5% bovine serum albumin (BSA), washed once with TBS containing 0.1% Triton X-100 (TBSt), and incubated with a primary antibody specific to DES at the proper dilution for 60 min at 37 °C. Cells were then rinsed three times with TBSt and incubated with the corresponding FITC or TRITC-conjugated secondary antibody (Santa Cruz Biotechnology, Inc.) for 60 min at 37 °C. Cells were rinsed three times with TBSt and incubated in 4, 6-diamino-2-phenylindole (DAPI) for 5 min to visualize nuclei. Cells were again rinsed three times with TBSt before observation.

### CRISPRi interference NCKAP5L transcription

To demonstrate the relationship between the expression of miR-2425-5p and NCKAP5L, three sgRNAs targeting different sites of the *NCKAP5L* (ID: 504364) promoter were designed. sgRNAs target sequences were designed as follows: N1: CACCGTGTCTGTGTAATCTGTAAG, N2: CACCGTCTCTGTTCTTGAGATGGA, and N3: CACCGTGTCTGTGTAATCTGTAAG. These oligonucleotides were synthesized, annealed, and ligated into the BbsI site under the hU6 promoter of pSPgRNA expression vector (Addgene, Middlesex, UK). MDSCs were plated in a 6-well plate 24 h before transfection. Cells were co-transfected with 2 mg of the dCas9 expression plasmid and 2 mg of the sgRNA expression plasmid. Total RNA was extracted after 48 h of transfection and SYBR green quantitative RT-PCR was performed to detect the expression of NCKAP5L and miR-2425. NCKAP5L detection primers were designed as CCAGCTCAGCACCTGATTTTGG (sense) and TCAGGCCCTGGGGATAAGTG (antisense).

### Statistical analysis

Data represent the mean ± SEM from three independent experiments and compared using ANOVA with post-hoc Tukeyʼs tests (SPSS, Inc., Chicago, IL, USA). One-sample t testing was used to perform the statistical significance test between groups. Differences were regarded as significant at a level of P < 0.05. *P < 0.05, **P < 0.01, NS: no significant difference.

## Electronic supplementary material

Original WB graphs in this manuscript
