# Supplementary material for: MiR-2425-5p targets RAD9A and MYOG to regulate the proliferation and differentiation of bovine skeletal muscle-derived satellite cells
Source: Sci Rep. 2017 Mar 24;7:418. doi: 10.1038/s41598-017-00470-8 (PMC5428422; doi:10.1038/s41598-017-00470-8)
Supplement: Supplementary file 1 — Original WB graphs in this manuscript [file 41598_2017_470_MOESM1_ESM.pdf]

**Manuscript title: MiR-2425-5p targets RAD9A and MYOG to regulate the proliferation and differentiation of bovine skeletal muscle-derived satellite cells**

**Authors:** Hui Li Tong, Run Ying Jiang, Wei Wei Zhang, Yun Qin Yan

**ID: SREP-16-28588A (B)**

**Information note:** We used polyclonal antibodies in our experiment. So you can see several blots in some WB graphs. Sometimes you can also see only one blot in some WB graphs because we cropped the NC membranes in WB process with the needs of experiments.

## Figure 2E

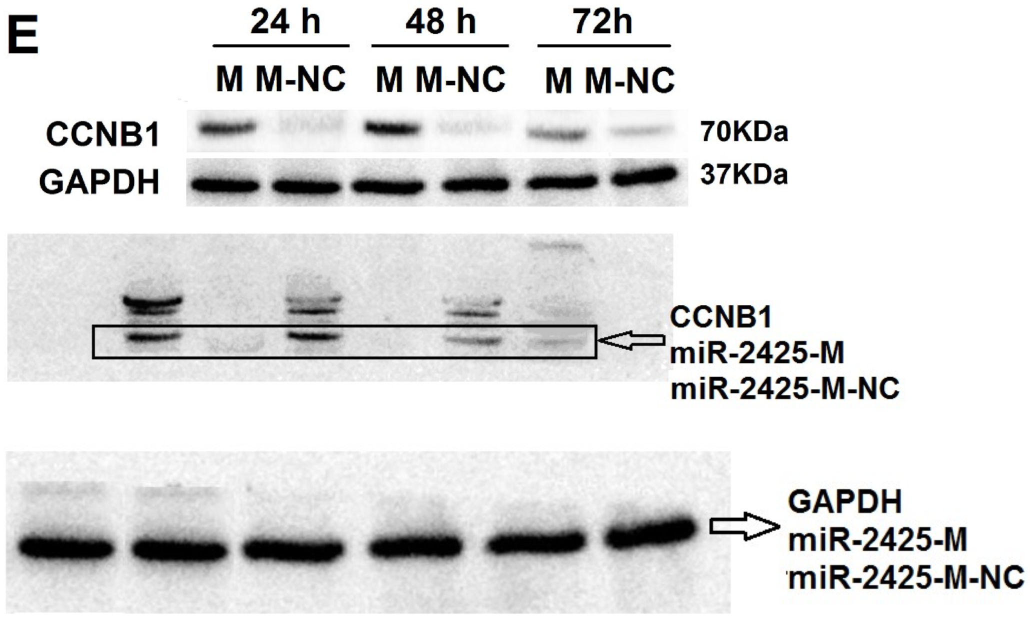


## Figure 2F

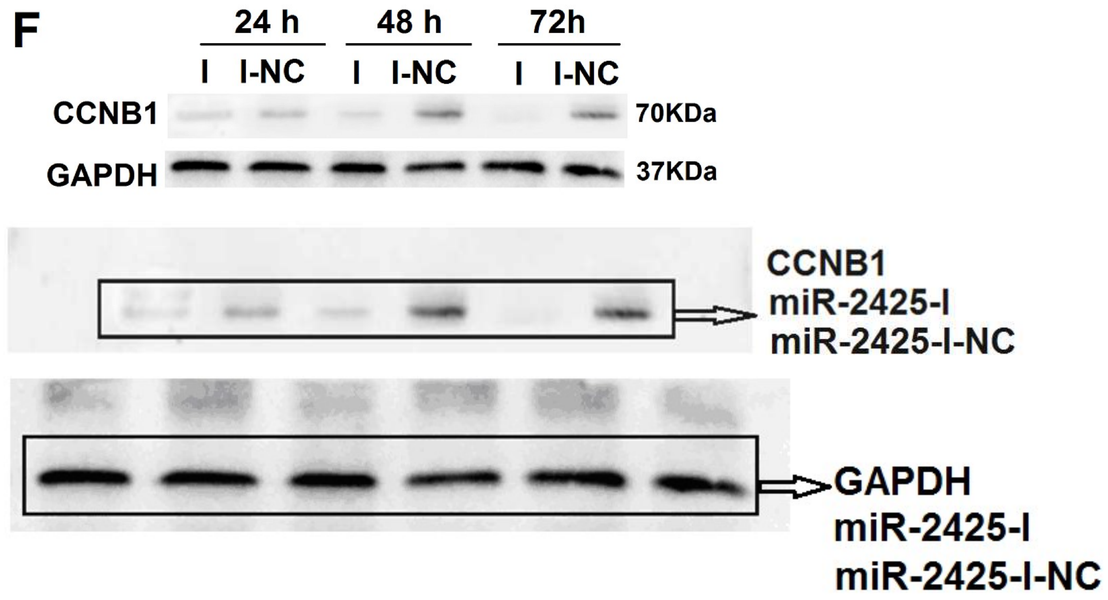

Figure 2G

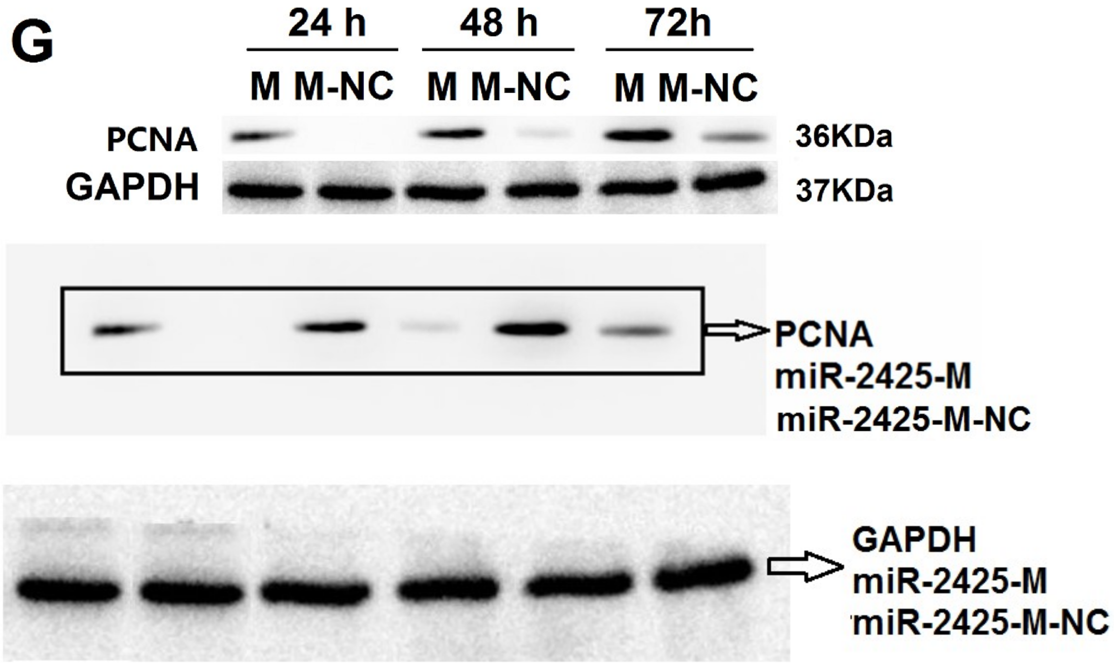


Figure 2H

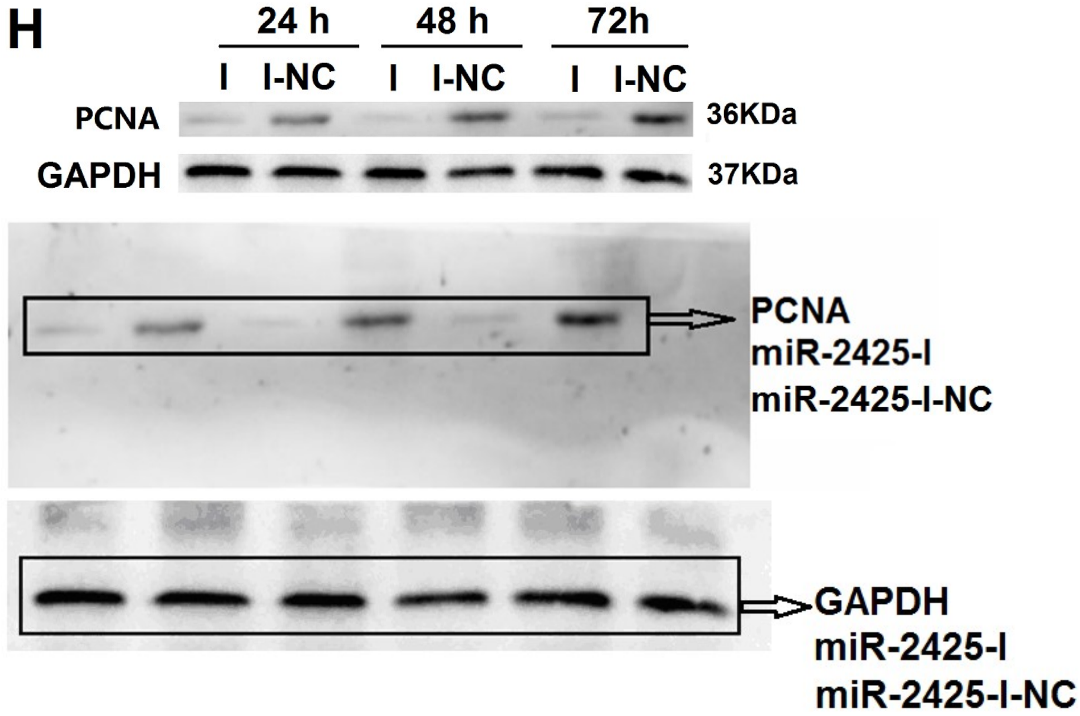

Figure 3C

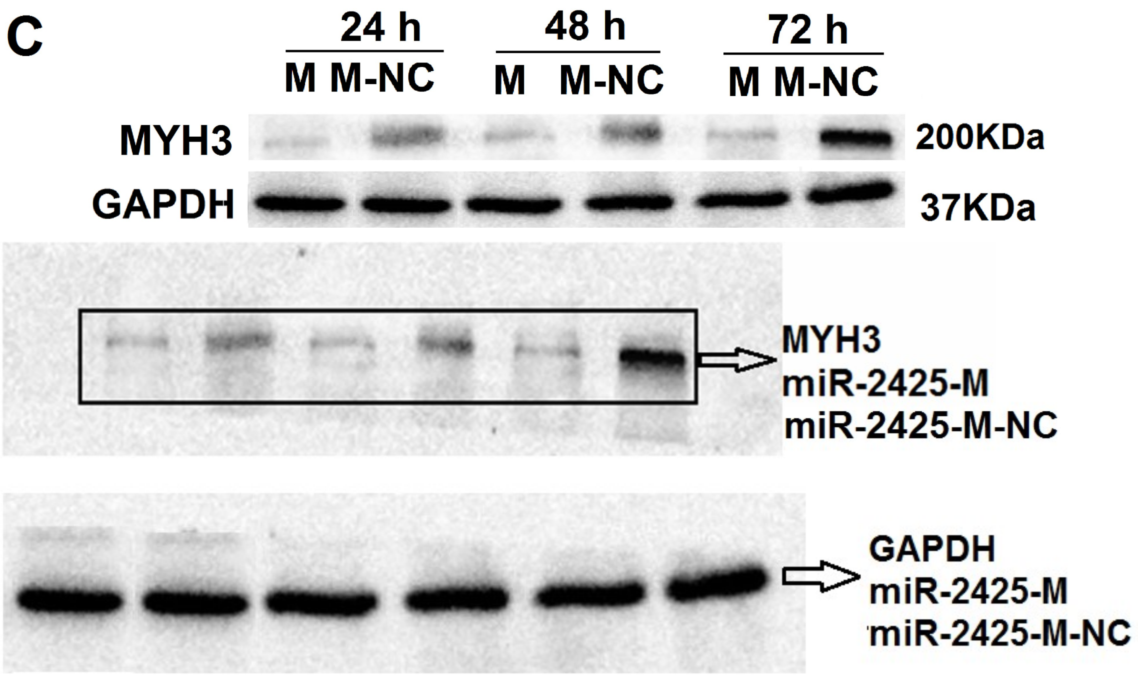


Figure 3D

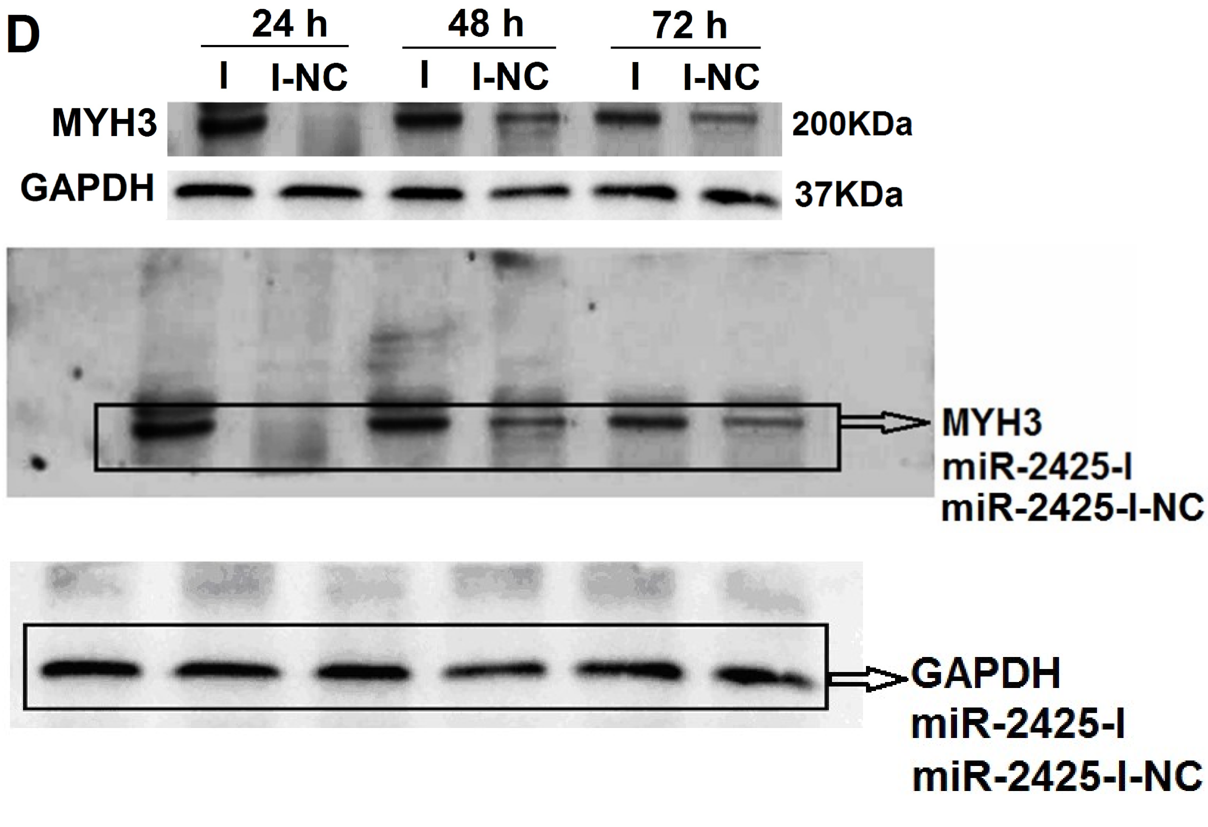

**Figure 4C**

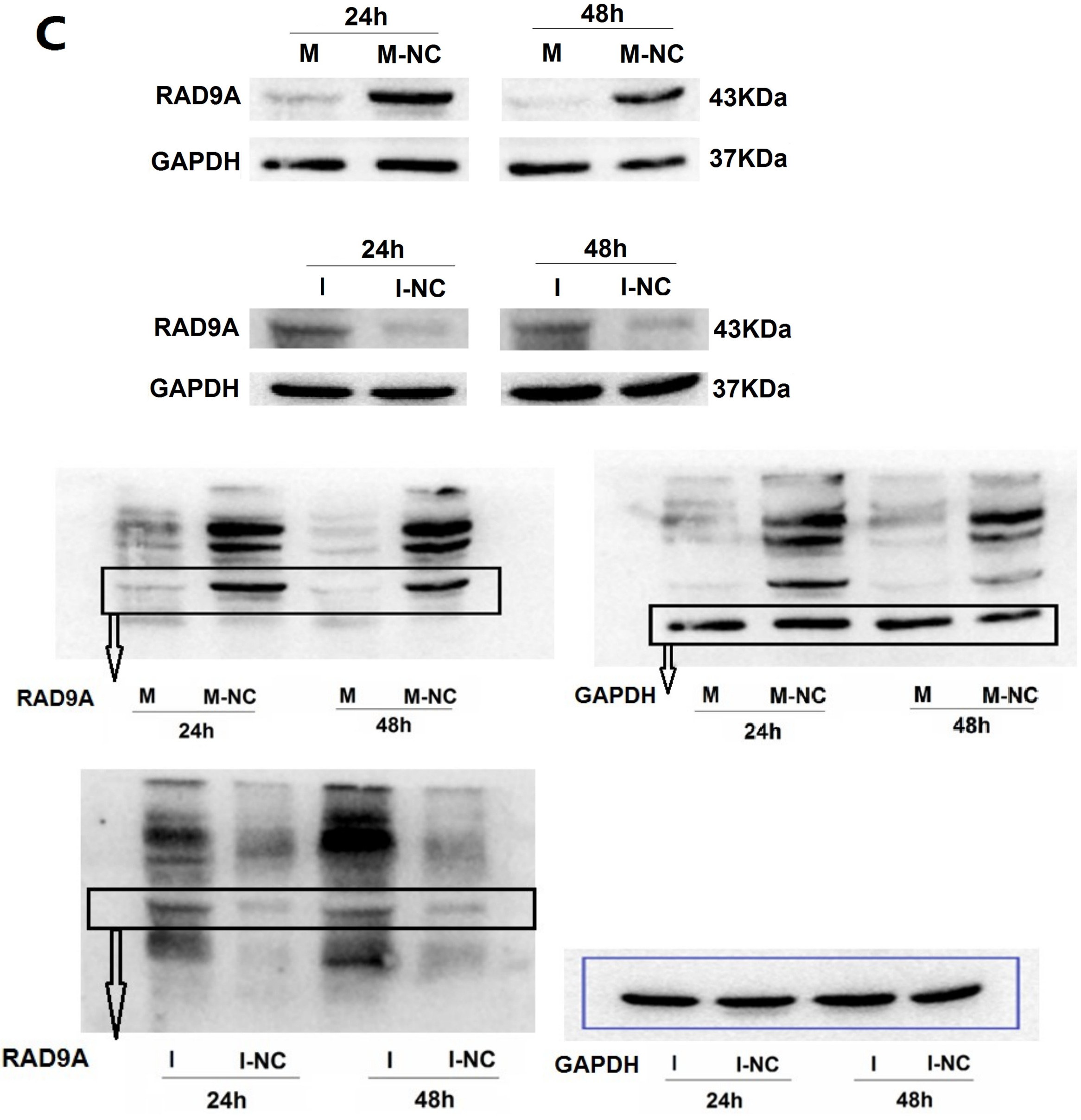
C
24h
48h
M
M-NC
RAD9A
GAPDH
43KDa
37KDa
I
I-NC
RAD9A
GAPDH
M
M-NC
I
I-NC

**Figure 4E**

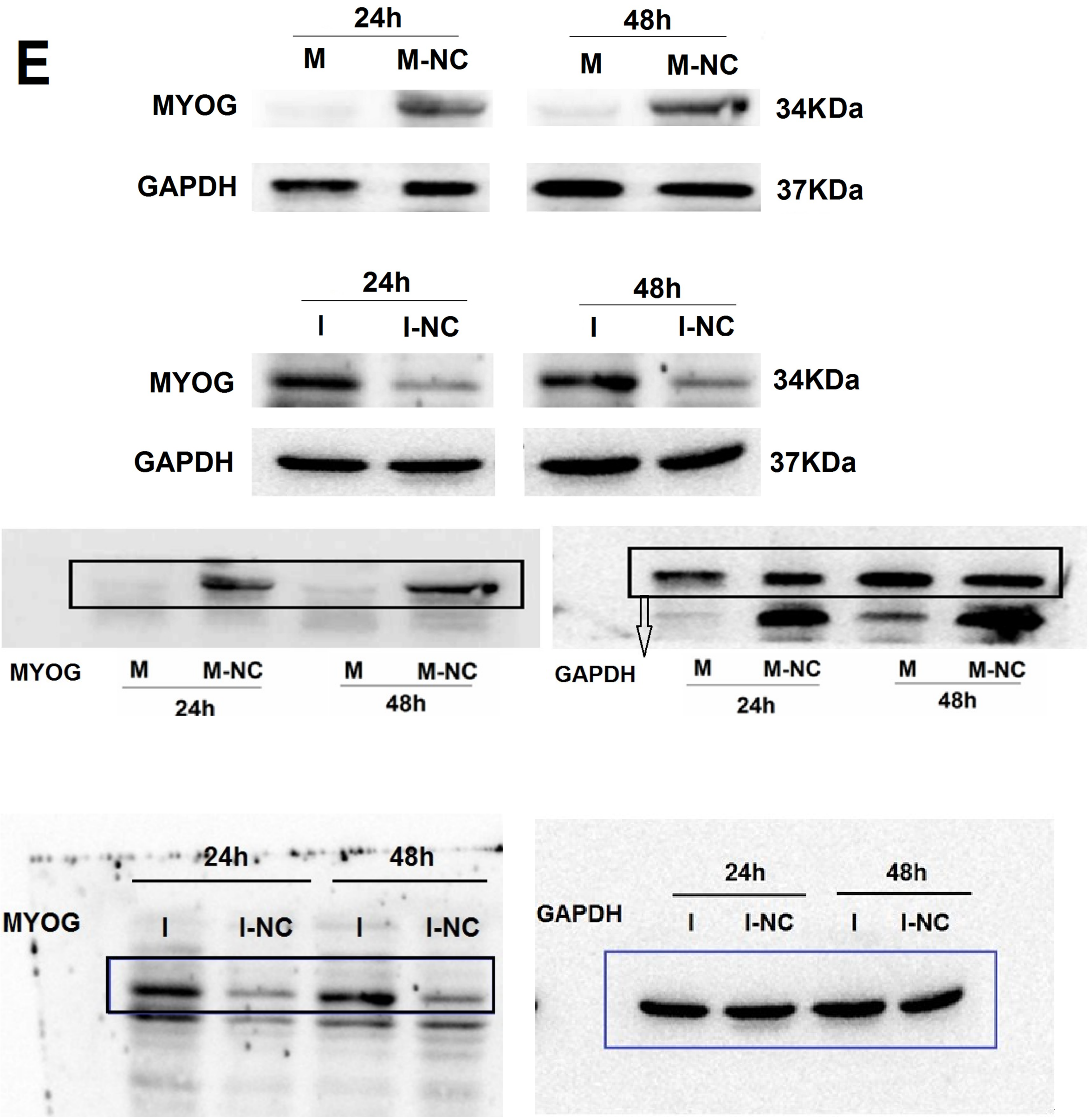

**Figure 5C**

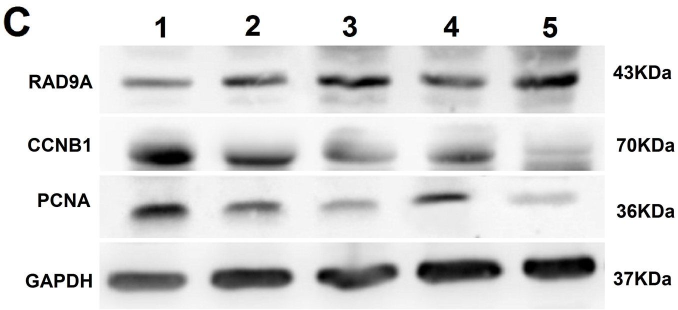
C
1
2
3
4
5
RAD9A
CCNB1
PCNA
GAPDH
43KDa
70KDa
36KDa
37KDa

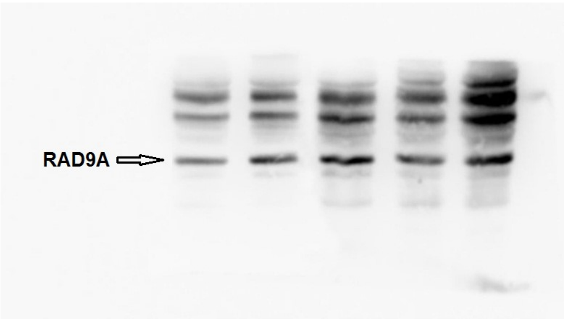
RAD9A

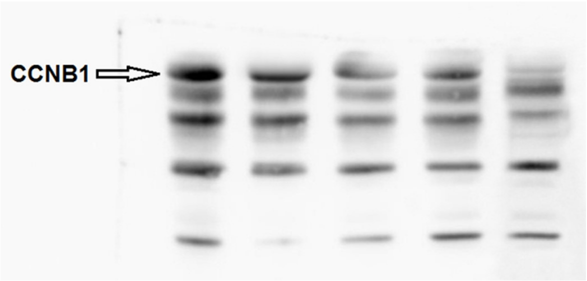
CCNB1

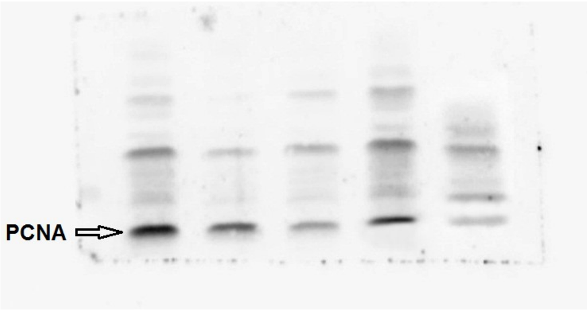
PCNA

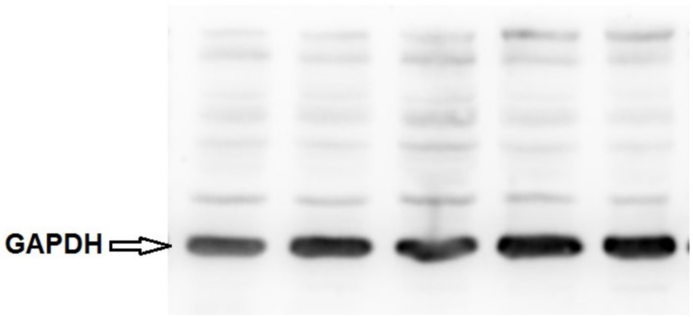
GAPDH
